# Can bladder endometriosis be hard to diagnose? A two-case report and literature review

**DOI:** 10.3389/fmed.2025.1607689

**Published:** 2025-09-04

**Authors:** Xingchen Li, Hong Li

**Affiliations:** Department of Radiology, People’s Hospital of Deyang City, Deyang, China

**Keywords:** bladder endometriosis, deep infiltrating endometriosis, partial cystectomy, pelvic MRI, case report

## Abstract

**Objectives:**

Bladder endometriosis (BE) is an uncommon form of deep infiltrating endometriosis (DIE). This report aims to present two cases of BE with markedly contrasting clinical histories and presentations to highlight diagnostic challenges and discuss management strategies.

**Methods:**

We describe the clinical presentation, diagnostic workup including pelvic magnetic resonance imaging (MRI), surgical management (laparoscopic partial cystectomy), histopathological findings, and short-term follow-up of two young women diagnosed with BE. Relevant literature is reviewed to contextualize the findings.

**Results:**

Both patients were accurately diagnosed preoperatively via MRI and underwent successful laparoscopic partial cystectomy, with histopathology confirming BE. Postoperative management involved a sequential protocol of gonadotropin-releasing hormone agonists (GnRH-a) followed by dienogest, which resulted in favorable short-term results, with no recurrence noted during follow-up.

**Conclusion:**

Diagnosing BE is often straightforward when typical clinical and imaging findings align. However, diagnostic delays are common due to the condition’s rarity and symptom overlap. Early diagnosis is crucial for achieving better outcomes. For women of reproductive age experiencing recurrent pelvic symptoms, even atypical ones, early pelvic imaging examinations are recommended. MRI plays a key role in diagnosing BE, guiding treatment decisions, and assisting with differential diagnosis. Enhancing awareness of BE among clinicians and radiologists is essential to expedite diagnosis and treatment.

## 1 Introduction

Endometriosis, the presence of endometrial-like tissue outside the uterine cavity, affects 6–10% of reproductive-aged women (1). Bladder endometriosis (BE), however, represents a rare manifestation, occurring in approximately 1% of patients with endometriosis (2). It is the most common site of urinary tract endometriosis (UTE), accounting for approximately 70–85% of all UTE cases (3). The condition’s rarity, coupled with often non-specific symptoms mimicking common urological or gynecological disorders, frequently leads to diagnostic delays. Early recognition and appropriate management are crucial to alleviate symptoms, prevent complications such as ureteral obstruction, and preserve quality of life.

This report details two cases of BE diagnosed and treated at our institution. The patients exhibited distinct clinical histories, symptom profiles, and lesion characteristics, offering insights into the varied nature of this condition. Both underwent successful laparoscopic partial cystectomy, confirming the diagnosis and achieving symptom resolution.

## 2 Case presentations

### 2.1 Case 1: presentation and initial findings

A 29-year-old married woman, gravida 4, para 1 (three prior induced abortions 4, 3, and 2 years previously), presented to the urology department with a 4-month history of urinary frequency, urgency, and dysuria, specifically exacerbated during menstruation. She also noted decreased menstrual flow but denied nausea, vomiting, gross hematuria, or fever. Her general physical examination was unremarkable. Laboratory findings were unremarkable, except for a urinalysis performed on day 7 of the patient’s cycle (post-menstruation), which revealed occult blood (2+) and microscopic hematuria (4.6 RBCs/HPF).

### 2.2 Case 1: imaging and diagnosis

**Figure 1:**
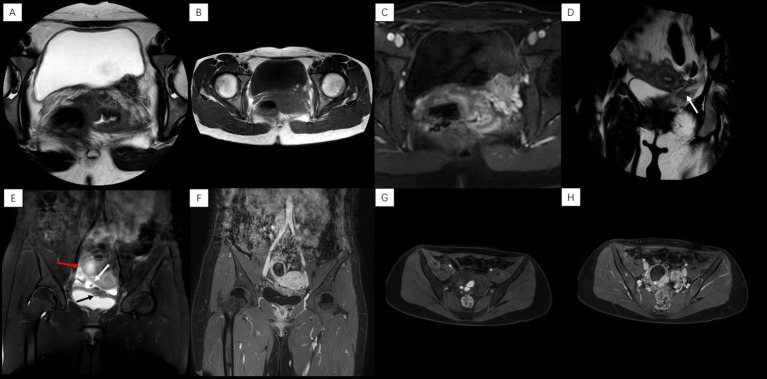
*Case 1*: **(A)** Axial T2WI: heterogeneous nodule, left posterior bladder wall. **(B)** Axial T1WI: high signal nodule within the lesion. **(C)** Axial enhanced T1WI: heterogeneous lesion enhancement. **(D)** Coronal T2WI: left ureteral orifice stenosis with hydroureter (arrow). *Case 2*: **(E)** Coronal T2WI: lesions in left superior bladder wall (black arrow), right vesicouterine space (white arrow, with hypointense nodule), and right ovarian region (red arrow). **(F)** Coronal enhanced T1WI: ring enhancement (ovarian lesion) and heterogeneous enhancement (other lesions). **(G)** Axial fat-suppressed T1WI: focal high signal in right ovarian cystic lesion. **(H)** Axial enhanced T1WI: ring enhancement, right ovarian lesion.

Pelvic MRI revealed a heterogeneous T2WI-hypointense mass on the left posterolateral bladder wall (Figure 1A), containing T1WI-hyperintense nodules suggestive of hemorrhage (Figure 1B). The lesion demonstrated marked heterogeneous enhancement on contrast-enhanced T1WI (Figure 1C) and involved the left ureteral orifice, causing stenosis and secondary hydroureter (Figure 1D). Notably, the remainder of the pelvis, including the uterus and ovaries, was unremarkable with no other evident lesions. However, based on the patient’s classic cyclical urinary symptoms and the characteristic MRI findings of hemorrhagic content, a preoperative diagnosis of bladder endometriosis was still strongly suspected.

### 2.3 Case 1: treatment and follow-up

**Figure 2:**
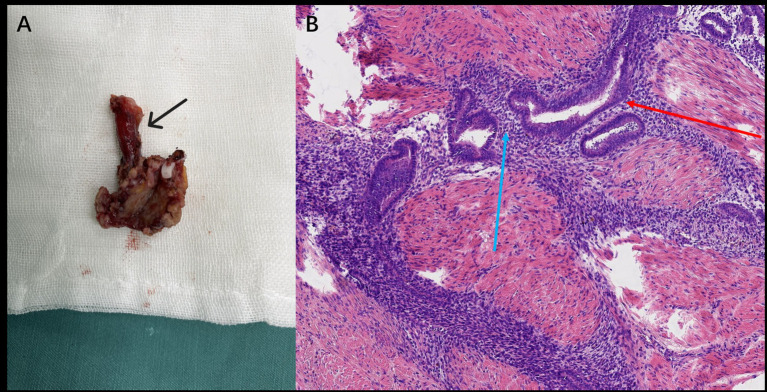
**(A)** Gross specimen (Case 1) showing the resected bladder lesion with the attached portion of the left ureter (black arrow). **(B)** Postoperative histopathology (Case 1, H&E stain, ×200) showing endometrial glands (red arrow) and stroma (blue arrow).

Following multidisciplinary discussion and confirmation of surgical suitability, the patient underwent laparoscopic partial cystectomy. Intraoperatively, a firm, vascular lesion measuring approximately 2.0 × 1.5 cm was identified on the left posterolateral bladder wall, directly encroaching upon the left ureteral orifice and infiltrating the distal ureter. The lesion was densely adherent to surrounding tissues. A partial cystectomy including the involved bladder wall and the left ureterovesical junction (requiring ureteral reimplantation) was performed (Figure 2A). Histopathological examination of the resected specimen confirmed the presence of endometrial glands and stroma within the bladder wall musculature, consistent with BE (Figure 2B). The patient recovered well and was discharged. Post-discharge, under the care of the gynecology clinic, she was started on a 6-month course of GnRH-a therapy (leuprolide acetate 3.75 mg intramuscularly monthly), with a planned transition to oral dienogest (2 mg daily) thereafter. During the 8-month follow-up, she reported experiencing no further urinary symptoms.

### 2.4 Case 2: presentation and initial findings

A 28-year-old unmarried woman with no history of childbirth or sexual activity presented to the urology department with a history of pelvic pain during menstruation for over 2 years and the incidental discovery of a bladder mass on ultrasound 1 week prior to admission. She reported seeking medical attention multiple times over the 2 years for cyclical pelvic pain without a definitive diagnosis. On this admission, her physical examination and routine blood tests were unremarkable. However, a urinalysis performed on day 13 of her cycle (mid-cycle) revealed occult blood (2+) and microscopic hematuria (14.9 RBCs/HPF).

### 2.5 Case 2: imaging and diagnosis

Pelvic MRI coronal T2WI showed multiple lesions in the left upper bladder wall, the right vesicouterine space, and the right ovarian region. The lesions in the right vesicouterine space and the left superior bladder wall are predominantly solid, exhibiting heterogeneous hypointense signals on T2WI. The lesion in the right ovarian region was cystic (approximately 4.7 × 4.1 cm in size) (Figure 1E). After enhancement, the lesion in the right ovarian region showed obvious ring-like enhancement, while the other lesions showed uneven and significant enhancement (Figure 1F). Axial non-contrast and contrast-enhanced T1-weighted imaging (T1WI) demonstrated a multilocular cystic lesion in the right ovary, containing non-enhancing hyperintense nodules within it (Figures 1G,H).

Prompted by the MRI finding of a complex adnexal mass, preoperative serum tumor markers were assessed. This revealed an isolated, mild elevation in cancer antigen 125 (CA-125) to 35.48U/mL (normal range: 0.00–32.40 U/mL), while other markers were within normal limits. The combination of these findings—multifocal pelvic lesions on imaging, a hemorrhagic ovarian cyst, and a mildly elevated CA-125—strongly supported the preoperative diagnosis of deep infiltrating endometriosis (DIE).

### 2.6 Case 2: treatment and follow-up

**Figure 3:**
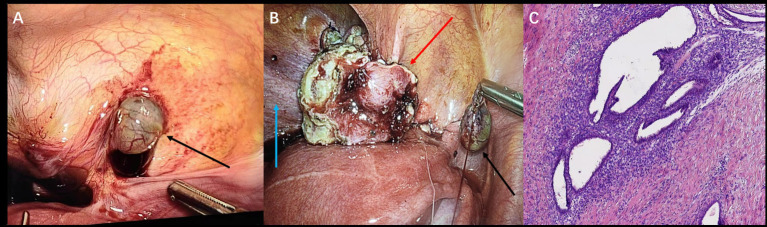
Laparoscopy (Case 2) **(A)** showing pelvic peritoneal lesions (black arrow) and **(B)** left superior bladder wall lesions (red arrow), with the bladder indicated (blue arrow). **(C)** Postoperative histopathology (Case 2, H&E stain, ×200).

Recognizing the disease’s multifocal nature and potential need for complex dissection, a combined laparoscopic approach by urology and gynecology teams was planned. Intraoperatively, findings included serosanguinous pelvic fluid, sigmoid adhesions, right ovarian cystic changes, and vesicouterine inflammatory tissue with cysts. A firm posterior bladder nodule, clear of the ureteral orifices, was confirmed and resected along with visible pelvic endometriotic implants (Figures 3A,B). However, considering the patient was nulliparous with a strong desire for future fertility, the ovarian cyst was intentionally left *in situ* for conservative management with postoperative hormone therapy. Histopathology confirmed endometriosis in all resected specimens (Figure 3C). The patient’s postoperative course was uneventful, and she was initiated on the same therapeutic protocol as the first patient—a 6-month course of GnRH-a followed by oral dienogest. By her 7-month follow-up, she reported a significant reduction in pelvic pain and the absence of any new symptoms.

## 3 Discussion

BE is characterized by the infiltration of endometriosis into the detrusor muscle and/or bladder epithelium, which may be partial or full thickness (4). The predominant understanding is that BE arises from infiltration by external peritoneal lesions. Consequently, the “multifocal” form, coexisting with other pelvic endometriotic lesions, is far more common (5). In contrast, the truly “isolated” form is exceptionally rare, reportedly accounting for only 10% of BE cases and affecting less than 0.1% of all women with endometriosis (6). Case 1 in this report appears to represent this uncommon isolated subtype, though the presence of small, difficult-to-detect non-vesical lesions cannot be definitively excluded.

The pathogenesis of BE remains debated, with four main hypotheses considered. The leading theory involves retrograde menstruation, where endometrial cells implant ectopically after refluxing onto pelvic sites, including the bladder wall (7). Alternatively, Müllerian remnant metaplasia suggests abnormal differentiation of embryonic tissues (8). Direct extension from uterine adenomyosis is another proposed mechanism (9). Lastly, iatrogenic implantation describes the transfer of endometrial tissue during procedures like cesarean sections (10).

Applying these theories to our cases: Patient 1’s history of three abortions might suggest surgically induced intrauterine pressure changes increasing retrograde flow as a potential cause. In contrast, Patient 2, with no history of surgery, childbirth, or sexual activity—a remarkably rare presentation for deep endometriosis—presented with multiple, adjacent lesions (ovary, bladder wall, posterior space), making retrograde menstruation a more likely explanation in her situation. This is supported by two key observations: first, the multifocal nature of her disease, with concurrent lesions in the ovary, vesicouterine space, and bladder, suggests a single “seeding” event. Second, and more importantly, the location on the posterior bladder wall is anatomically significant. This surface forms the anterior boundary of the vesicouterine space, a dependent area where refluxed endometrial cells naturally pool. Therefore, the posterior bladder wall represents a classic and direct implantation site, making retrograde menstruation the most plausible pathogenic explanation in her specific clinical context.

The clinical presentation of BE is notoriously heterogeneous, a factor that critically contributes to diagnostic delay. While 30% of patients are asymptomatic, discovered incidentally, the remaining 70% present with a wide spectrum of lower urinary tract symptoms (LUTS) (11). Typically, this can manifest as cyclic LUTS, such as cyclic dysuria/painful urination, frequency/urgency, or, in rare cases, cyclic hematuria (12). However, a large systematic review of 390 symptomatic women highlights a more fragmented reality: dysuria was the most frequently cited symptom, yet it was reported by only 27.18% of patients. A nearly identical proportion (27.95%) experienced only generic LUTS, while hematuria was observed in a mere 10.77% (13). Critically, symptoms may be entirely non-cyclical (14). Furthermore, these symptoms may be influenced by lesion size and location, adding another layer of variability (7).

Our two cases starkly illustrate this clinical spectrum. Case 1, with her classic LUTS exacerbated by her menstrual cycle, represents the more recognizable presentation that can lead to a prompt suspicion of BE. In contrast, Case 2 exemplifies the more common diagnostic pitfall. Her presentation with only non-specific pelvic pain, in the complete absence of LUTS, aligns with a large cohort of patients whose bladder pathology is either clinically silent or masked by more dominant symptoms from coexisting DIE. Her two-year diagnostic delay is a direct consequence of this non-specific presentation and underscores the profound impact on patient well-being. This evidence strongly supports the need for a high index of suspicion and early recourse to pelvic imaging, especially when the classic urinary clues are absent.

This need for prompt, accurate imaging is underscored by the diagnostic challenge of a solid bladder wall mass. The differential diagnosis is broad, but the clinical priority is always the exclusion of malignancy. In our cases, the solid, enhancing nature of the mass—and particularly the local invasion causing ureteral obstruction in Case 1—creates a significant imaging overlap with its most critical mimic: urothelial carcinoma (UC) (15). The diagnostic pivot, however, was the presence of T1-hyperintense foci—a signature of subacute hemorrhage characteristic of endometriosis but rare in UC (15, 16). This unique composite of a T2-hypointense stroma and hemorrhagic spots is key to navigating the differential.

This hemorrhagic signature also helps distinguish BE from other mimics. Malignancies like leiomyosarcoma and metastases, along with benign entities such as leiomyomas, hemangiomas, and inflammatory masses, typically lack this key feature (15, 16). This distinction is particularly crucial for mimics like leiomyomas and amyloidosis, which can present with T2-hypointense components that resemble the fibrotic stroma of BE, yet they are differentiated by the absence of the classic hemorrhagic signal (15). While the multifocality in Case 2 could suggest metastases, the consistent bleeding within each lesion strongly supported a unified endometriotic origin. Ultimately, a confident diagnosis relied on synthesizing this specific imaging profile with the broader clinical context—namely, the cyclical symptoms and corroborating lesions seen in Case 2—to reliably distinguish BE from its mimics.

Transvaginal ultrasound (TVS) and MRI are both important imaging examinations for evaluating BE. TVS has become the first-line examination technique because of its accuracy, safety, and cost-effectiveness (17). The specificity of TVS for diagnosing BE can reach 100%; however, its sensitivity is relatively low, especially for small endometriotic nodules (18). Furthermore, TVS is an invasive examination, and the examination process may cause patient discomfort. Given that TVS possesses relatively high diagnostic accuracy, MRI is considered a second-line imaging technique because of its higher cost, but it has higher contrast resolution in evaluating bladder wall layers and tissue characteristics, which is beneficial for differential diagnosis. Furthermore, MRI, by virtue of its multiplanar imaging capability, can clearly display the relationship between BE and surrounding structures (e.g., uterus, ureters), and can concurrently evaluate other deep infiltrating endometriosis lesions, which is particularly important for surgical planning (17). In patients whose preoperative ultrasound is negative despite symptoms suggesting BE, an MRI examination may prove helpful for establishing the correct diagnosis (19). In this report, both patients underwent pelvic MRI examination and obtained accurate diagnoses. MRI’s precise display of the lesion extent was an important prerequisite for the successful surgery of these two patients, especially for the second patient involving multiple pelvic lesions. Therefore, we believe that for BE patients requiring surgery, undergoing an MRI examination is highly necessary, as isolated BE is rare and concurrent lesions must be evaluated. This conclusion, however, requires confirmation by studies with larger cohorts.

The management strategy for BE should be individualized, primarily depending on the severity of symptoms, lesion characteristics, patient age, and fertility intentions (17). Medical treatment, such as hormone therapy (including combined oral contraceptives (COCs), progestogens like dienogest, or GnRH-a), can effectively relieve pain and urinary tract symptoms (17, 20). However, medical treatment is primarily suppressive rather than curative; its effect may be limited, particularly for deep infiltrating lesions containing significant fibrous tissue, and symptoms and lesions often recur after stopping the medication (8). Therefore, for BE patients with significant symptoms, complete surgical excision is the preferred method (21). Laparoscopic partial cystectomy is the standard procedure, allowing for complete excision while maximally preserving bladder function and facilitating concurrent treatment of other pelvic endometriosis (21, 22). In contrast, transurethral resection (TUR) is generally insufficient for deep lesions and carries higher risks of recurrence and perforation (21).

Postoperative hormone therapy is often recommended to suppress potentially residual microscopic disease and lower the risk of recurrence, yet high-quality evidence supporting its universal benefit remains limited (20, 23). In a comparative study, Fedele et al. reported that while both GnRH-a and COCs induced regression of bladder lesions, GnRH-a led to a more pronounced regression (24). More recently, research has shown that using a short course of a GnRH-a as a postoperative “bridge” before initiating long-term dienogest (DNG) therapy effectively mitigates early adverse bleeding, thereby improving patient compliance and overall quality of life (25). Furthermore, a 2021 guideline from Burghaus et al. (26) recommends a six-month, rather than a three-month, regimen of a GnRH-a to significantly reduce recurrence risk.

Given that both of our patients were at high risk for recurrence, we opted for a proactive strategy to maximally mitigate this risk: a short course of GnRH-a therapy (involving 6 injections) followed by continuous DNG. This regimen has yielded favorable short-term outcomes, although validation of its long-term benefits in larger cohorts is required.

This report is limited by its small sample size and short follow-up duration. Long-term outcomes regarding symptom control, recurrence, and fertility require further monitoring.

## 4 Conclusion

In general, the diagnosis of bladder endometriosis (BE) is not difficult when typical clinical symptoms and imaging manifestations are combined. However, its rarity and overlapping clinical symptoms may delay clinical diagnosis and adversely affect the physical and mental health of patients. The early identification of BE is crucial for improving patient outcomes. Therefore, women of reproductive age experiencing recurrent pelvic symptoms, even atypical ones, should receive pelvic imaging examinations as early as possible. MRI plays a key role in diagnosing BE and guiding treatment decisions, aiding in differential diagnosis. Ultimately, fostering greater vigilance for BE among clinicians and radiologists is necessary to expedite diagnosis and treatment, thereby substantially improving patients’ long-term health and quality of life.

## Data Availability

The original contributions presented in the study are included in the article/supplementary material, further inquiries can be directed to the corresponding author.
